# PreMLS: The undersampling technique based on ClusterCentroids to predict multiple lysine sites

**DOI:** 10.1371/journal.pcbi.1012544

**Published:** 2024-10-22

**Authors:** Yun Zuo, Xingze Fang, Jiayong Wan, Wenying He, Xiangrong Liu, Xiangxiang Zeng, Zhaohong Deng

**Affiliations:** 1 School of Artificial Intelligence and Computer Science, Jiangnan University, Wuxi, China; 2 School of Artificial Intelligence, Hebei University of Technology, Tianjin, China; 3 Department of Computer Science and Technology, National Institute for Data Science in Health and Medicine, Xiamen Key Laboratory of Intelligent Storage and Computing, Xiamen University, Xiamen, China; 4 School of Information Science and Engineering, Hunan University, Changsha, China; Indian Institute of Technology Mandi - Kamand Campus: Indian Institute of Technology Mandi, INDIA

## Abstract

The translated protein undergoes a specific modification process, which involves the formation of covalent bonds on lysine residues and the attachment of small chemical moieties. The protein’s fundamental physicochemical properties undergo a significant alteration. The change significantly alters the proteins’ 3D structure and activity, enabling them to modulate key physiological processes. The modulation encompasses inhibiting cancer cell growth, delaying ovarian aging, regulating metabolic diseases, and ameliorating depression. Consequently, the identification and comprehension of post-translational lysine modifications hold substantial value in the realms of biological research and drug development. Post-translational modifications (PTMs) at lysine (K) sites are among the most common protein modifications. However, research on K-PTMs has been largely centered on identifying individual modification types, with a relative scarcity of balanced data analysis techniques. In this study, a classification system is developed for the prediction of concurrent multiple modifications at a single lysine residue. Initially, a well-established multi-label position-specific triad amino acid propensity algorithm is utilized for feature encoding. Subsequently, PreMLS: a novel ClusterCentroids undersampling algorithm based on MiniBatchKmeans was introduced to eliminate redundant or similar major class samples, thereby mitigating the issue of class imbalance. A convolutional neural network architecture was specifically constructed for the analysis of biological sequences to predict multiple lysine modification sites. The model, evaluated through five-fold cross-validation and independent testing, was found to significantly outperform existing models such as iMul-kSite and predML-Site. The results presented here aid in prioritizing potential lysine modification sites, facilitating subsequent biological assays and advancing pharmaceutical research. To enhance accessibility, an open-access predictive script has been crafted for the multi-label predictive model developed in this study.

## Author summary

Proteins undergo a variety of post-translational modifications (PTMs) after synthesis, such as lysine modifications, which significantly influence their structure and function. These modifications of lysine are known to regulate physiological processes, including the inhibition of cancer cell growth, the delay of aging, the regulation of metabolic diseases, and the improvement of depressive disorders. Abnormal modifications are closely associated with the occurrence and progression of a multitude of diseases. Therefore, the identification and comprehension of these modifications are of paramount importance for biological research and drug development. A multitude of studies have focused on a single type of lysine modification, with prediction methods for multiple lysine modification sites being relatively scarce. In this research, a novel multi-label prediction model named PreMLS has been developed for the simultaneous identification of four lysine modifications: methylation, acetylation, crotonylation, and succinylation. The imbalance issue in the dataset was addressed utilizing the ClusterCentroids undersampling algorithm, following which a predictive model, PreMLS, was constructed using a CNN to forecast multiple lysine modification sites. Compared to existing models, this new approach has significantly enhanced the accuracy and reliability of the predictions.

## Introduction

Within biological systems, the covalent attachment of a diverse array of small chemical moieties to proteins subsequent to their synthesis is termed post-translational modification (PTM) [1,2] This phenomenon constitutes an essential regulatory phase for protein function and is critical for cellular processes. PTMs include a spectrum of modifications, such as phosphorylation, methylation, and acetylation, which synergistically shape the architecture and functionality of proteins. Dysregulation of PTMs is intricately linked to the etiology and progression of a multitude of diseases; hence, the precise mapping of PTM sites is paramount for elucidating the molecular underpinnings of biological mechanisms and pathogenesis [3–6]. Lysine residue modifications, characterized by covalent attachments of chemical groups to the lysine side chains in proteins, have been implicated in the modulation of physiological processes, including gene transcription, depression, HIV latency, and oncogenesis [7]. Additionally, acetylation has been correlated with conditions such as obesity, diabetes, and metabolic disorders [8]; elevated succinylation levels are posited to expedite ovarian senescence [9] and have been significantly linked to gastric cancer development [10]; and the perturbation of lysine methylation in transcription factors can suppress the proliferation of cancer cells [11]. Collectively, the identification of lysine modification sites is pivotal for investigating their implications in metabolic pathways, genetic expression, and a spectrum of diseases, including cancer, as well as for the advancement of therapeutic interventions.

Mass spectrometry is instrumental in the domain of post-translational modifications (PTMs) of proteins, providing an exceedingly potent tool for the surveillance and identification of such modifications. It facilitates a broad analysis of the proteome, progressively elucidating the comprehensive profile of PTMs within protein populations. This holistic analytical capacity grants insights into the distribution and dynamics of various modifications across the proteome. Additionally, mass spectrometry affords quantitative assessment of modification levels, enabling the investigation of modifications’ alterations under diverse physiological conditions or pathological states. Such quantitative analyses can uncover the interplay between modifications and biological processes, offering vital insights for diagnostic and therapeutic endeavors. Nevertheless, despite its numerous merits in detecting PTMs, mass spectrometry equipment demands specialized expertise for operation and maintenance and commands a high price. The financial outlay for acquisition, upkeep, and utilization of mass spectrometers can impose a considerable financial strain on many research facilities. Moreover, prior to mass spectrometry analysis, samples are often subjected to a multifaceted preparatory phase, encompassing processes such as extraction, purification, and fractionation. These procedures can entail significant investment in time and labor, as well as the potential for sample degradation or contamination. Furthermore, mass spectrometry is not without its drawbacks, including finite sensitivity and resolution constraints.

In contrast to conventional mass spectrometry methodologies, machine learning presents several compelling benefits, including cost-effectiveness, technical efficiency, extensive predictive capabilities, and precision [12–19]. Consequently, the application of machine learning approaches for the detection of post-translational modification sites has become increasingly vital. Within the domain of identifying single types of lysine modification sites, in 2017, Wang et al. [20] harnessed the power of support vector machines (SVM) and its enhanced algorithms, devising local sequence kernels and Gaussian interaction profile kernels. These kernels adeptly capitalized on local sequence information and modification-site relationships, amalgamating multiple kernels to train SVM for the prediction of phosphorylation sites. Kernel-based methodologies can attain superior performance by integrating biological insights through an aptly chosen kernel function, which projects data points into a higher-dimensional space, thereby facilitating effective prediction. In 2020, Ju et al. [21] introduced a novel predictive tool, leveraging Chou’s five-step protocol and the General Pseudo component with k-interval amino acid pairs, to formulate a lysine formylation site prediction method termed CKSAAP_FormSite. A biased SVM was employed to surmount the class imbalance challenge in formylation site prediction, with experimental outcomes attesting to its commendable performance. In 2021, Huang et al. [22], inspired by the shortcomings of DeepSuccinyl-Site and the loss of semantic residue relationships, merged long short-term memory (LSTM) networks with convolutional neural networks (CNN) into a deep learning framework for succinylation site prediction. This approach captured the semantic relationships embedded within succinylation sequences, exemplifying the profound advantages of deep learning frameworks over traditional machine learning in data classification endeavors. In 2022, OOdeyomi et al. [23] proposed a deep learning architecture for the prediction of succinylation sites on lysine residues, comparing its efficacy with state-of-the-art deep learning models and other conventional machine learning techniques for succinylation. The performance metrics highlighted a commendable equilibrium between computational velocity and classification accuracy. Advancing into 2024, Gao et al. [24] introduced a multi-view neural network, MVNN-HNHC, for the identification of human non-histone crotonylation sites. This methodology integrated multi-view encoded features and deployed multi-channel neural networks across various feature types, thereby garnering more insightful information to enhance the comprehension of discriminative attributes. The findings demonstrated the method’s robust predictive prowess concerning non-histone crotonylation.

In the domain of discerning multiple lysine modification sites, a model termed iPTM-mLys was developed by Qiu et al. [25] in 2016 to identify four distinct lysine modifications. Nonetheless, this approach compartmentalized the overarching problem into four discrete binary classification challenges, which were addressed individually and then synthesized, inadvertently overlooking the interplay between PTMs at various sites. In 2021, the research collective headed by Ahmed unveiled a duo of models, iMul-kSite [26] and predML-site [27], aimed at the identification of multiple lysine modification sites, which similarly deconstructed the multi-site recognition issue into a series of binary classification problems. iMul-kSite initiates by purging redundant samples from the predominant class through an assessment of sequence coupling information’s rigidity, subsequently integrating F-Test evaluations and incremental feature selection methodologies to refine feature representation, and culminates with the application of support vector machine (SVM) technology for dichotomous discrimination among sample categories. Conversely, the predML-site model leverages a cost-sensitive SVM for binary classification, enhancing the management of imbalanced datasets by apportioning variable costs to misclassifications across different categories.

Crosstalk, wherein multiple PTMs on identical or disparate proteins exert mutual influence and collaborate, has been demonstrated to synergistically modulate a plethora of physiological processes through lysine post-translational modifications [28–30]. Nonetheless, the majority of prior investigative methodologies have concentrated on the forecasting of singular modification types, with computational models predominantly endowed with the capacity to predict a solitary PTM variety. A novel multi-label predictive model is introduced in this investigation, leveraging an expanded dataset as a reference to prognosticate concurrently four distinct lysine residue modifications: methylation, acetylation, crotonylation, and succinylation. Regarding feature extraction, In this research, the extant multi-label position-specific triad amino acid propensity (MLPSTAAP) algorithm has been adopted, which is adept for multi-label prediction as delineated in Zuo’s study [31]. It is a well-documented fact that imbalanced datasets frequently harbor some significant yet infrequent categories (minority classes), in stark contrast to other categories (majority classes) that boast a substantial sample volume. The class imbalance within datasets has a tendency to bias classification algorithms towards recognizing the more populous categories, concurrently diminishing their discernment for less frequent categories [32]. To surmount this class imbalance predicament, the ClusterCentroids undersampling technique is utilized by PreMLS. Given the proficiency of convolutional neural networks (CNN) in capturing both localized features and overarching patterns, they amplify the model’s comprehension and portrayal of sequence data. The classification framework of PreMLS is constructed using a CNN. It has been demonstrated that commendable predictive performance has been achieved by PreMLS in both training and testing datasets, with a significant outperformance over counterparts in multi-label prediction, such as iMul-kSite and predML-Site.

## Results

### Determining the undersampling ratio of Cluster Centroids

In our study, the training set is composed of 11 classes with a distribution ratio of 9279:710:600:454:561:252:360:88:153: 454:73. It is evident that the first class significantly outnumbers the others, accounting for over 71% of the total samples. The disparity in the number of samples among the remaining classes is not as pronounced. Consequently, Under-sampling has been exclusively applied to the first class to address this imbalance.

The under-sampling ratio denotes the ratio of the number of samples post-under-sampling to the original number of samples. Different under-sampling ratios have distinct impacts on the representativeness of the post-sampled data and the training of the model. When a lower under-sampling ratio is employed, the loss of original samples is substantial, leading to a significant reduction in the range of data representation. This diminishes the capacity of the sampled data to adequately represent the original data distribution, causing biases in model predictions and an inability to accurately capture data features. Additionally, a low sampling ratio may result in insufficient sample sizes, which can lead to underfitting as the model fails to learn the data feature representations adequately.

On the other hand, a higher under-sampling ratio means that the post-sampled dataset retains more information from the majority class, maintaining strong representational capabilities. However, due to the extreme imbalance in the original dataset’s class distribution, a lower under-sampling effort may still fail to eliminate the dataset’s class imbalance. In such instances, the model may become over-fitted to the characteristics of the majority class during training, resulting in a bias towards the prediction of these classes and potentially leading to overfitting. This does not achieve the intended effect of under-sampling.

It is clear that selecting a moderate under-sampling ratio is crucial. This ensures that the post-sampled dataset maintains adequate data representation, significantly mitigating the class imbalance issue and preventing the model from becoming ineffective by predicting all samples as the first class.

Explanations for the abbreviations used are provided in Table 1: A, C, M, and S represent acetyllysine, crotonyllysine, methyllysine, and succinyllysine, respectively, indicating four types of post-translational lysine modifications. The absolute accuracy rate of multi-label prediction (Absolute-True) indicates the proportion of samples for which the model’s predicted labels match the true labels completely. For these absolutely accurate predictions, the model neither predicts additional labels not present in the sample nor omits any labels that the sample possesses.

**Table 1:** Absolute accuracy of 11 types of samples under 5-fold cross-validation under different undersampling ratios (%).

Ratio	A	C	M	S	AC	AM	AS	CM	ACM	ACS	ACMS
1	98.76	27.18	0.00	1.98	11.69	75.94	71.94	2.95	93.73	10.53	100.00
0.8	98.02	32.93	0.00	3.22	6.02	75.22	74.00	9.32	87.32	8.72	100.00
0.5	98.03	33.27	0.00	2.60	10.37	75.78	67.28	4.55	93.86	9.12	100.00
0.3	96.62	43.01	0.00	1.76	8.27	67.49	65.72	6.14	87.58	9.16	100.00
0.15	89.62	56.85	0.00	1.10	5.28	70.52	66.11	50.23	92.94	10.09	100.00
0.13	89.39	56.62	0.00	1.67	4.85	71.47	67.17	56.82	92.68	10.70	100.00
**0.1**	**87.82**	**59.72**	**0.00**	**3.13**	**6.70**	**72.03**	**65.44**	**68.86**	**90.07**	**10.09**	**100.00**
0.07	78.71	66.25	0.00	6.30	4.67	72.27	68.83	76.59	87.45	10.04	100.00
0.05	75.85	48.37	0.00	2.07	3.78	52.27	53.83	2.73	79.74	6.61	100.00

From Table 1, it is evident that as the under-sampling ratio decreases, the model’s absolute accuracy rate for samples in categories containing only label C (crotonyllysine) and those containing both C and M (crotonyllysine and methyllysine) significantly improves. The absolute accuracy rate for samples with only acetyllysine modifications exhibits a gradual decline, while other categories do not show significant differences. Considering the necessity for a sufficient sample size in neural network training and to ensure that the under-sampled dataset improves the model’s generalization for minority classes without compromising its predictive power for the majority class, a final under-sampling ratio of 0.1 has been selected.

### Ablation experiment

Ablation experiments evaluate the impact of specific model components by progressively removing or altering them, with the aim of assessing the contribution of each component and thereby elucidating the effectiveness of the model’s components.

In this research, we systematically dissected the model’s components through a phased ablation strategy, encompassing four experimental setups: 1. Training the Convolutional Neural Network (CNN) on the original dataset without under-sampling; 2. Implementing a random under-sampling strategy on the original data prior to CNN training; 3. Applying a cluster-centroid-based under-sampling method followed by CNN training on the resultant dataset. 4. Apply the NearMiss1 and NearMiss2 undersampling methods, followed by training on the processed dataset. The findings, as delineated in Table 2, reveal that the model’s performance, post-random under-sampling, is consistently inferior across metrics when juxtaposed with the non-resampled and Cluster-Centroids-enhanced models. The performance of the ClusterCentroids undersampling algorithm is also significantly superior to that of NearMiss-1 and NearMiss-2. Given the negligible variance in performance metrics between the standalone CNN and the augmented CNN with Cluster-Centroids across five evaluation criteria, we conducted a detailed analysis of the absolute accuracy rates for the 11 categories to discern subtleties in their predictive capabilities.

**Table 2:** Five-fold cross-validation results under different sampling methods (%).

Methods	Aiming	Coverage	Accuracy	Absolute True	Absolute False
CNN	84.44	**80.28**	**79.05**	**72.37**	12.17
CNN+RandomUnder	80.43	75.90	73.29	67.46	13.70
CNN+ClusterCentroids	**84.54**	78.26	77.00	72.34	**11.83**
CNN+Nearmiss1	74.88	67.74	65.07	58.92	17.26
CNN+Nearmiss2	80.42	72.40	69.80	63.68	14.91

Upon meticulous statistical analysis, we observed that in the absence of sampling, the model’s predictive accuracy for the 11 classes of lysine modification data was as follows: 99.92%:0.00%:0.00%:0.00%:10.77%:0.00%:0.00%:0.00%:21.57%:0.31%:40.00%; Upon the application of the ClusterCentroids under-sampling method, the model’s predictive accuracy for the 11 classes of lysine modification data was as follows: 85.33%:56.62%:0.00%:5.81%:8.20%:74.82%:68.61%:98.64%:89.15%:14.14%:100.00%.

**Fig 1:**
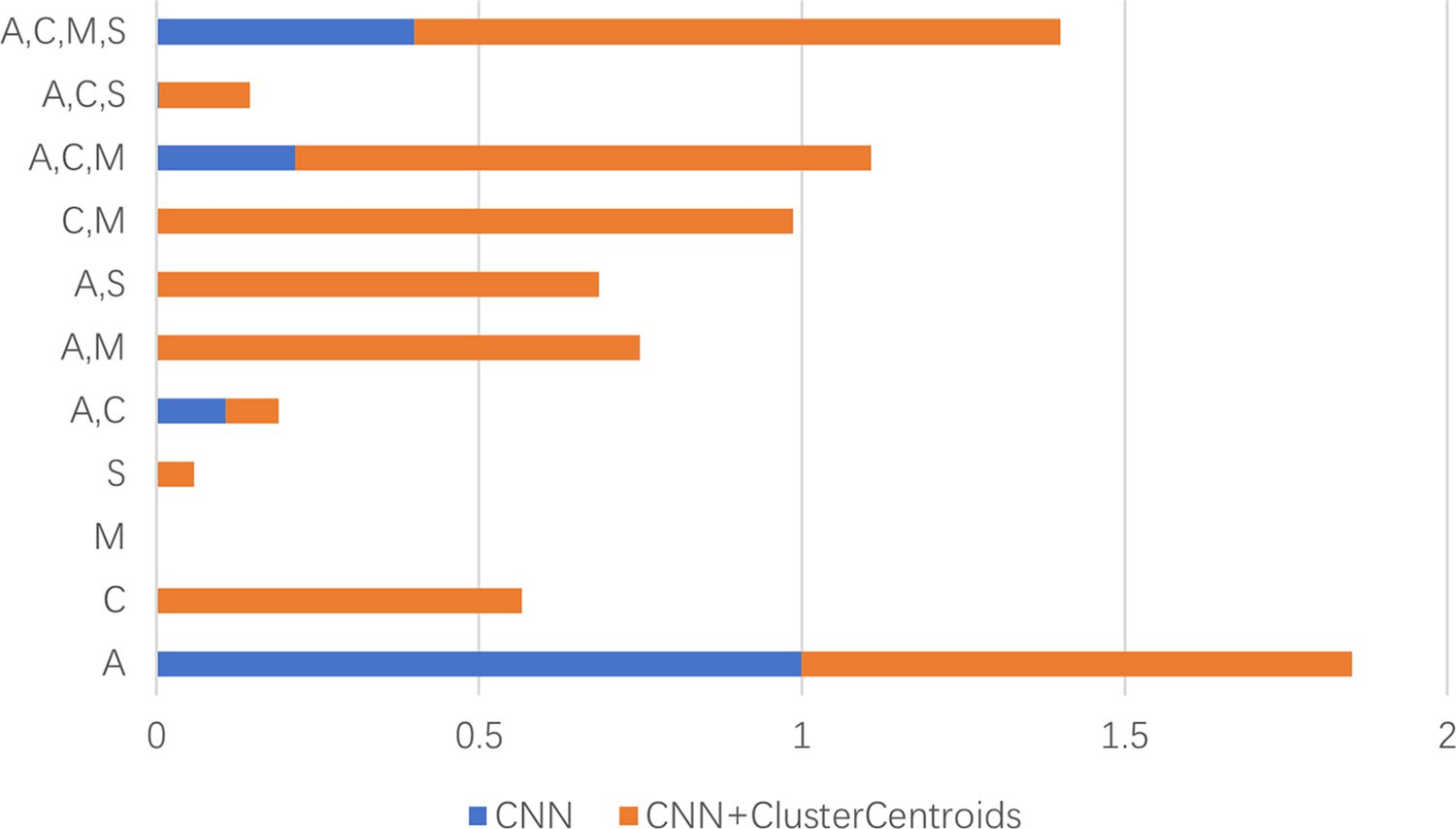
Comparison of the absolute accuracy of CNN and CNN+ClusterCentroids in eleven types of lysine modification. A, C, M, S represent acetyllysine, crotonyllysine, methyllysine, and succinyllysine respectively, which are four types of post-translational modifications of lysine.

It is evident that the model trained on the original dataset exhibits a high degree of fit for the first class, which is the majority class, but performs poorly in predicting other classes. Notably, six classes were not correctly predicted at all, indicating that the model has become overfitted to the first class due to class imbalance. In contrast, training on the dataset following under-sampling with the ClusterCentroids method has resulted in a significant improvement in the model’s predictive capabilities across all classes except the first. This suggests that the under-sampled dataset has achieved effective balance, thereby improving the model’s predictive power across multiple classes. To further elucidate the efficacy of the model developed in this study, we have visualized the absolute accuracy statistics of the two models, which have comparable performance across five evaluation metrics, on the 11 classes. The detailed results are presented in Fig 1.

### Comparison with existing prediction models

#### Five-fold cross-validation comparison

Initially, a five-fold cross-validation [33–36] comparison experiment was conducted using the proposed model PreMLS against three existing prediction models: iMul-kSite, mLysPTMpred [37], and iPTM-mLys [25], with the results presented in Table 3. The following scores were achieved by PreMLS on the five evaluation metrics: 86.31%, 80.18%, 78.51%, 73.54%, and 10.94%, placing it third among the four models.

**Table 3:** Comparison of different models based on five-fold cross-validation result (%).

Models	Aiming	Coverage	Accuracy	Absolute True	Absolute False
iMul-kSite	93.18	96.13	92.70	88.77	2.97
mLysPTMpred	84.82	86.56	83.73	79.73	6.66
iPTM-mLys	69.78	74.54	68.37	60.92	13.40
PreMLS	86.31	80.18	78.51	73.54	10.94

Although performance of PreMLS in the five-fold cross-validation experiment was not particularly prominent, when comparative experiments were conducted on an independent test dataset, it was observed that our model also exhibited a certain degree of generalization ability, significantly outperforming iMul-kSite. This indicates that while iMul-kSite may have good predictive performance on the training set, its performance on the test set suggests a tendency towards overfitting and weak generalization capabilities. In contrast, PreMLS has demonstrated a favorable balance between fitting and generalization capabilities.

### Comparison with existing models on independent test datasets

**Fig 2:**
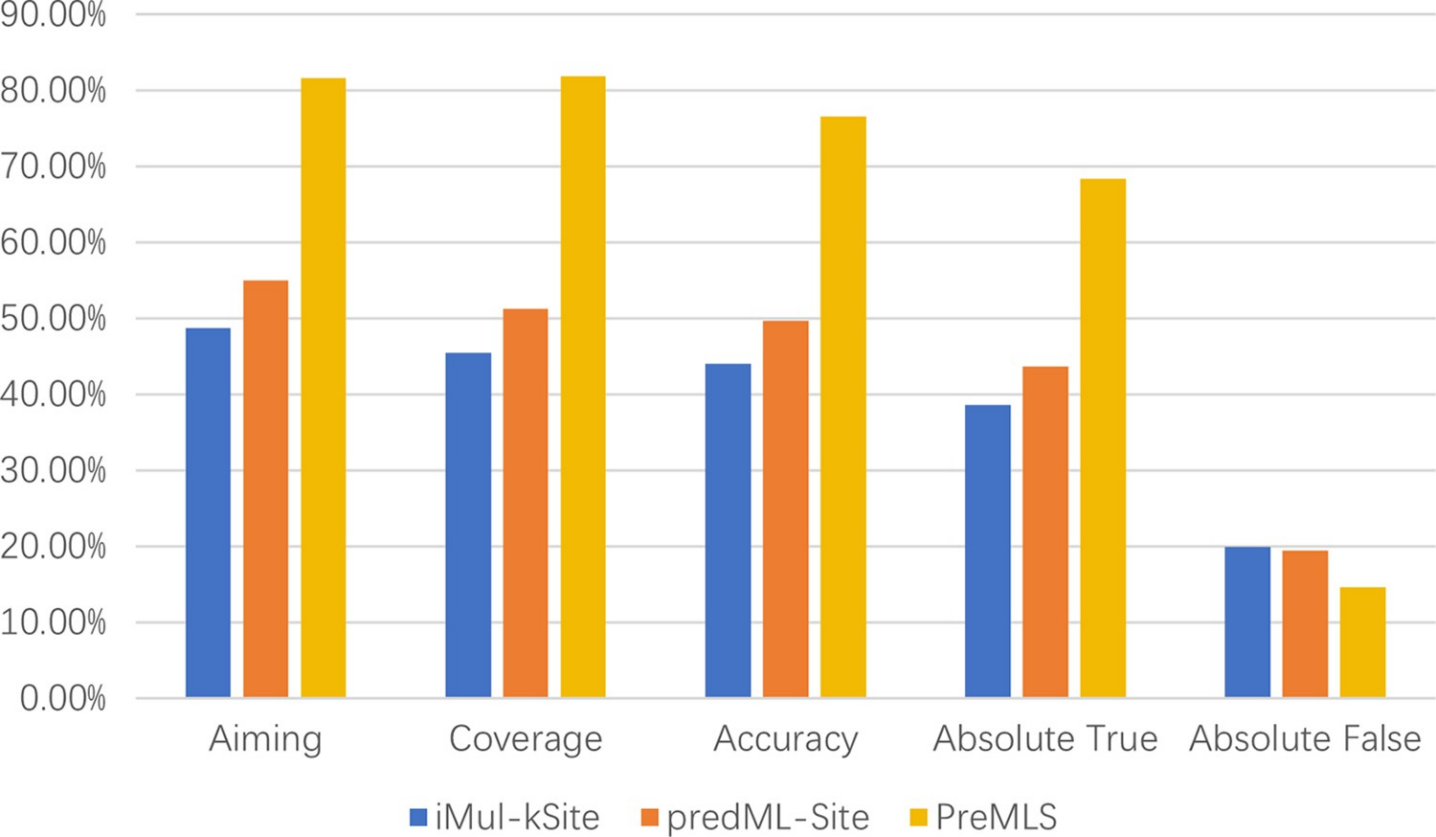
Comparison of the performance of different prediction tools on the test set.

As depicted in Fig 2, a comparative analysis was conducted between PreMLS and the readily accessible web servers’ iMul-kSite and predML-Site, using the independent test dataset specifically curated for this research. The existing predictive tools, iMul-kSite and predML-Site, employ a strategy that decomposes the multi-label prediction challenge into a series of binary classification tasks. The process entails the segmentation of the dataset for each label into two distinct categories: samples with the label and those without, generating a binary classification of positive and negative instances. Addressed through an algorithm enhanced with the principles of Support Vector Machine (SVM), these binary classifications are then integrated to construct a comprehensive multi-label prediction model. The comparative analysis demonstrated that PreMLS shows a significant improvement of approximately 30% in both Aiming and Coverage metrics, along with an enhancement of over 25% in Accuracy and Absolute-True, and a modest5% decrement in Absolute-False. These results underscore the superior performance of our model over the existing tools on the test set. Notably, upon examining the precision of predicted labels, it was observed that iMul-kSite and predML- Site demonstrate a markedly lower accuracy in identifying samples with multiple lysine modification sites. Specifically, these models tend to predict only a subset of the multiple labels present in the majority of multi-label samples. Conversely, PreMLS demonstrated a higher absolute accuracy and was capable of effectively predicting multi-label samples, as evidenced by the data. The reasons for the superior predictive performance of PreMLS over iMul-kSite and predML-Site have been analyzed: iMul-kSite and predML-Site decompose the multi-label prediction problem into a series of binary classification tasks, thereby neglecting the interplay between post-translational modifications (PTMs) at different sites. In contrast, PreMLS is adept at leveraging this information. Furthermore, PreMLS employs the ClusterCentroids under-sampling algorithm, effectively mitigating the issue of data imbalance. These findings highlight the advantage of constructing a dedicated multi-label prediction model, which significantly outperforms the binary classification ensemble approach in multi-label prediction tasks.

#### SHAP explain ability analysis

To elucidate the workings of our predictive model and the derivation of its outputs, The SHAP (SHapley Additive exPlanations) method was employed to interpret the model’s prediction process. SHAP, rooted in game theory, is a model-agnostic technique that estimates the influence of individual features on the prediction outcome, assigning each feature a corresponding explanatory score. The computation of SHAP values leverages the Shapley value theory, an equitable allocation mechanism that ascribes the final output—such as a prediction—to the contribution of each feature. The specific influence of individual features on the model’s predicted outcomes is evaluated by examining all possible combinations of features. In the context of deep learning models, Shapley values quantify the contribution of each input feature to the model’s predictive performance, with higher absolute values indicating a more significant influence on the outcome.

**Fig 3:**
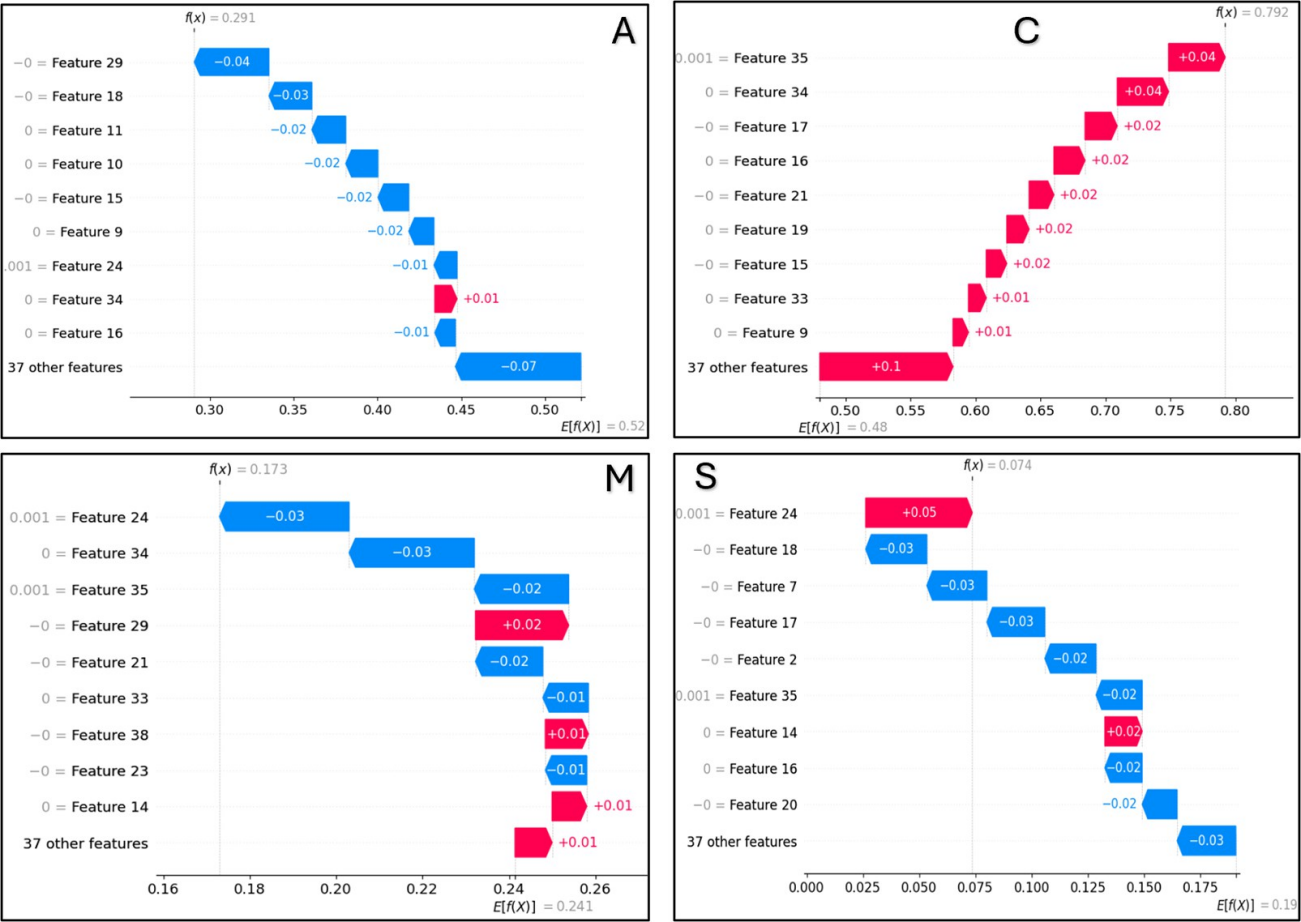
SHAP analysis of a sample.

A SHAP analysis was conducted on a sample with the predictive label (0, 1, 0, 0), where the labels (A, C, M, S) denote "acetyllysine," "crotonyllysine," "methyllysine," and "succinyllysine," respectively—abbreviations for four types of post-translational lysine modifications. We calculated the Shapley values for the features of this sample with respect to the different labels, as illustrated in Fig 3.

Fig 3 is interpreted as follows: the vertical axis represents the feature index, while the horizontal axis displays the Shapley values for different features. For the A label prediction, E[f(x)] = 0.52 indicates the average predicted value for A across the dataset. Red bars represent positive contributions to the prediction value, while blue bars denote negative contributions. For instance, as shown in Fig 3, a positive contribution of 0.01 is attributed to Feature34, while a negative contribution of 0.04 is attributed to Feature29. The bars are sorted in descending order of their absolute impact on the prediction value, highlighting key factors such as Features 29, 18, 11, 10, 15, 9, 24, 34, and 16 that predominantly influence the prediction of label A. This visual representation allows us to discern which features predominantly influence the prediction of the four distinct labels and to appreciate the differences in the absolute influence of various features on the prediction of the same label.

#### Predictor and user guide

To present the experimental outcomes of our study in a visually engaging manner and to offer an interactive experience, we’ve developed a demo interface for a predictive tool, accessible at https://github.com/xzfang00/Protein-multi-classification-main/tree/main. This interface is designed with user experience at its core, offering a clean and intuitive layout for easy data prediction. It also incorporates a robust technical framework and data processing pipeline to ensure accurate and swift predictions. The tool is crafted to be a practical aid for interpreting experimental data. The usage of the predictive tool is as follows:

Step 1: Launch the predictor, select the feature-extracted test data in.mat format; choose the trained model file in.pth format.

Step 2: upon completion of file selection click on "Predict" to initiate the prediction process.

In the prediction results page, the column "Sample" sequentially indicates our sample numbers. The "Results" column refers to the predicted labels for the samples, while the "Targets" column represents the actual labels of the samples. The predicted labels A, C, M, and S correspond to A: acetyllysine, C: crotonyllysine, M: methyllysine, and S: succinyllysine, respectively. The above is a brief demonstration of how to use this prediction tool. For more detailed instructions and information on other related files of the prediction tool, please refer to our GitHub documentation at Protein-multi-classification/README.md.

## Discussion

Three main components are encompassed by this study: data preprocessing, the construction of a convolutional neural network (CNN), and experimental analysis. Initially, the dataset was partitioned into training and testing sets at a ratio of 7:3. Protein sequences were encoded into numerical vectors using a multi-label positional-specific scoring matrix (PSSM) algorithm, followed by one-hot encoding for the multiple labels of the samples. To mitigate class imbalance, the ClusterCentroids framework was utilized for undersampling of the majority classes, and an appropriate undersampling ratio was selected through experimentation.

The feasibility of employing CNNs for biosequence analysis was elucidated, complemented by a schematic representation of the adopted neural network architecture. Comparative experiments demonstrated that our method significantly outperforms existing approaches, such as iMul-kSite and predML-Site, in multi-label prediction performance. Ablation studies further showcased the efficacy of undersampling, revealing that centroid-based undersampling is more representative of the original data distribution and reduces information loss compared to random undersampling.

In conclusion, constructing a direct multi-label prediction model is essential for predicting multiple lysine modification sites, as it exhibits superior predictive performance compared to binary classification ensemble models. Moreover, class imbalance can be effectively alleviated by appropriate resampling of data, which enhances the model’s predictive and generalization capabilities for minority classes and reduces overfitting in majority classes. The key points of this study are summarized in Box 1.

**Box 1. The key points of this study**

**Key Points**

- Lysine post-translational modifications (PTMs) are a ubiquitous biological phenomenon that holds significant value in biological research and drug development. However, current research has largely been focused on single types of modifications, with the interplay between different PTMs at multiple sites being overlooked. To address this gap, PreMLS has been introduced in our study, capable of directly predicting multiple lysine modification sites.
- In our approach, class imbalance is adeptly mitigated by employing a ClusterCentroids undersampling strategy, which leverages MiniBatchKmeans. This strategy enhances model generalizability across prevalent classes and is notably efficient in terms of memory consumption.
- The bespoke convolutional neural network (CNN) architecture engineered in our study adeptly harnesses the sequential dependencies, outperforming existing models such as iMul-kSite and predML-Site in multi-label predictive accuracy.
- Model interpretability analysis based on SHAP values indicates that PreMLS constructed in this study is effective.

Despite the model’s promising predictive capabilities, there is scope for enhancement: 1. Algorithm Refinement: The ClusterCentroids method, while mitigating class imbalance, may omit vital data. Future work should be conducted to investigate alternative resampling strategies that preserve features and achieve better class representation. 2. Model Architecture Optimization: The convolutional and pooling layers within the model show signs of overfitting on the test set. Further research should incorporate regularization techniques, fine-tune pooling parameters, and consider Dropout to bolster generalization. 3. Data Engineering Advancement: The current data engineering approach is basic and affects the uniformity of the model’s multi-class prediction. Diverse feature extraction techniques should be employed in subsequent studies to improve class representation. 4. Limitations of the CNN Architecture: Although superior performance is demonstrated by PreMLS in predicting lysine modification sites compared to several existing models, the CNN architecture is found to have certain limitations in capturing long-range dependencies. Some modification sites in protein sequences may be influenced by amino acid residues that are distantly located, and CNNs are considered less effective than Recurrent Neural Networks (RNNs) or Transformer architectures in extracting such global contextual information. Additionally, the fixed window size of CNNs is seen to limit their ability to handle irregular sequence lengths and complex data structures, potentially resulting in reduced prediction accuracy for certain modification sites. Therefore, the future research consider the incorporation of, or substitution with, other deep learning architectures, such as LSTM networks or self-attention mechanisms, to allow for better capturing of long-range dependencies and complex features within sequences.

## Materials and methods

### Dataset description

To construct a robust statistical prediction model necessitates a high-quality dataset. In this study, the dataset construction method employed by Zuo [31] was utilized, sourcing all human protein sequences from CPLM4.0, a database cataloging lysine PTMs. CPLM4.0 consolidates data from 10 public repositories, meticulously reorganizing and mapping lysine PTM sites to UniProt, thereby filtering out redundancies. Our dataset comprised 18,978 human protein sequences, categorized by PTM type: acetyllysine (11,863), crotonyllysine (2,151), methyllysine (3,152), and succinyllysine (1,812). To construct a dataset suitable for both training and testing, a meticulous and rigorous strategy was adopted, with specific steps as follows:

Step 1: Sequence Truncation and Verification: The 18,978 sequences were precisely truncated to extract peptides with confirmed central lysine modifications (including acetylation, crotonylation, methylation, and succinylation). Employing a 24-window sliding window centered on lysine, we generated 49-amino-acid fragments to capture contextual features influencing modification site prediction.

Step 2: Post-initial processing, sequences were classified into 15 categories based on four modification states. To ensure data balance and representativeness, a minimum threshold of 60 sequences per category was established. This filtering yielded 11 categories for model training and evaluation ensuring the balance and representativeness of each category’s data and to avoid bias due to small sample sizes.

This rigorous process ensured dataset diversity and reliability, providing a solid foundation for our statistical prediction model. Our eleven data categories are as follows:

$$\left\{ \begin{array}{l} \phi (1)=\phi_{1}(\mathrm{acetyllysine})\\ \phi (2)=\phi_{2}(\mathrm{crotonyllysine})\\ \phi (3)=\phi_{3}(\mathrm{methyllysine})\\ \phi (4)=\phi_{4}(\mathrm{succinyllysine})\\ \phi (5)=\phi_{5}(\mathrm{acetyllysine}\cap \mathrm{crotonyllysine})\\ \phi (6)=\phi_{6}(\mathrm{acetyllysine}\cap \mathrm{methyllysine})\\ \phi (7)=\phi_{7}(\mathrm{acetyllysine}\cap \mathrm{succinyllysine})\\ \phi (8)=\phi_{8}(\mathrm{crotonyllysine}\cap \mathrm{methyllysine})\\ \phi (9)=\phi_{9}(\mathrm{acetyllysine}\cap \mathrm{crotonyllysine}\cap \mathrm{methyllysine})\\ \phi (10)=\phi_{10}(\mathrm{acetyllysine}\cap \mathrm{crotonyllysine}\cap \mathrm{succinyllysine})\\ \phi (11)=\phi_{11}(\mathrm{acetyllysine}\cap \mathrm{crotonyllysine}\cap \mathrm{methyllysine}\cap \mathrm{succinyllysine}) \end{array}\right.$$ (1)

Data Category Description: ∅(1) = ∅_1_ (acetyllysine) refers to protein sequences where the central lysine is exclusively modified by acetylation. ∅(5) = ∅_5_(acetyllysine∩crotonyllysine) refers to protein sequences that contain both acetylation and crotonylation modifications. The symbol "∩" indicates that the samples in this category have multiple post-translational modifications. To eliminate redundant peptide fragments in the data, the resulting sample counts for each of the 11 categories are as follows: ∅(1):39938,∅(2):2462,∅(3):3107,∅(4):1019,∅(5):4695,∅(6):972,∅(7):1220,∅(8):138,∅(9):277,∅(10):1161,∅(11):125.

As can be seen from the above, the first category has a sample count of 39,938, the second category has a sample count of 2,463, and so on. To ensure that the performance estimation of the multi-label prediction model is not overestimated due to sequence redundancy and homology, in this study, the CD-HIT [38] program was selected, with a threshold of 0.4 set to eliminate potential homologous sequences and redundant samples. For the obtained 11 categories of data, 70% of each category was randomly selected as the training data, and the remaining 30% as the test data. The resulting non-redundant split datasets are as follows:

Training set: ∅(1):9279,∅(2):710,∅(3):600,∅(4): 454,∅(5):561,∅(6):252,∅(7):360,∅(8):88,∅(9):153,

∅(10):454,∅(11):73。Testing set: ∅(1):4062,(2):304,∅(3):257,∅(4):194,∅(5):240,∅(6):107,∅(7):154,∅(8):42,∅(9):73,∅(10):191,∅(11):36.

### Multi-label labeling method

For the 11 categorized classes, a hybrid approach combining one-hot encoding and numerical mapping vectors was employed to formulate the multi-label datasets. Each class was assigned a unique numerical identifier, with the first class labeled "1," the second "2," and so forth. One-hot encoding was applied to convert categorical data into a binary format suitable for machine learning analysis. This technique represents each category with a distinct binary vector, where a single "1" denotes the category’s index, and "0" fills all other positions. This binary representation disassociates any inherent ordinal relationships, ensuring a more scientifically rigorous and rational measurement within the feature space. The encoding process is succinctly as follows:

Step 1: Determine the Number of Categories: Initially, ascertain the total number of distinct categories within the discrete feature set, assuming there are n different categories.

Step 2: Assign Integer Encoding for Each Category: Sequentially encode each category with an integer ranging from 1 to n. These integer codes can be arbitrary as long as they uniquely identify each category.

Step 3: Construct One-Hot Encoding: For each sample, the feature is represented by a vector of length n, where all elements are set to 0 except for the position corresponding to the category index, which is set to 1. For instance, when processing the categorical attribute of a specific sample, if its attribute value is at the j-th position, the j-th position in the vector will be marked as 1.

The research subject of this paper is lysine methylation, crotonylation, acetylation, and succinylation, hence there are four discrete feature labels for the samples. The constructed four-dimensional one-hot encoding vector corresponding to the labels is (acetyllysine, crotonyllysine, methyllysine, succinyllysine).

Since the protein sequences in this paper may undergo lysine methylation and acetylation simultaneously, or other combinations of lysine PTMs, the corresponding one-hot encoding vector for a sample may contain one or more "1"s. The positions of "1"s correspond to the indices of the PTM categories that the sample has undergone. For example, for a protein sequence where the central lysine contains only acetylation, its category is ∅(1) = ∅_1_ (acetyllysine), and the corresponding one-hot encoding is (1,0,0,0). For a protein sequence sample that contains both acetylation and crotonylation, its category is ∅(5) = ∅_5_ (acetyllysine∩crotonyllysine) and the corresponding one-hot encoding is (1,1,0,0). The one-hot encodings corresponding to each category are as follows:

$$\left\{ \begin{array}{l} \phi_{1}(\mathrm{acetyllysine})=(1,0,0,0)\\ \phi_{2}(\mathrm{crotonyllysine})=(0,1,0,0)\\ \phi_{3}(\mathrm{methyllysine})=(0,0,1,0)\\ \phi_{4}(\mathrm{succinyllysine})=(0,0,0,1)\\ \phi_{5}(\mathrm{acetyllysine}\cap \mathrm{crotonyllysine})=(1,1,0,0)\\ \phi_{6}(\mathrm{acetyllysine}\cap \mathrm{methyllysine})=(1,0,1,0)\\ \phi_{7}(\mathrm{acetyllysine}\cap \;\mathrm{succinyllysine})=(1,0,0,1)\\ \phi_{8}(\mathrm{crotonyllysine}\cap \mathrm{methyllysine})=(0,1,1,0)\\ \phi_{9}(\mathrm{acetyllysine}\cap \mathrm{crotonyllysine}\cap \mathrm{methyllysine})=(1,1,1,0)\\ \phi_{10}(\mathrm{acetyllysine}\cap \mathrm{crotonyllysine}\cap \mathrm{succinyllysine})=(1,1,0,1)\\ \phi_{11}(\mathrm{acetyllysine}\cap \mathrm{crotonyllysine}\cap \mathrm{methyllysine}\cap \mathrm{succinyllysine})=(1,1,1,1) \end{array}\right.$$ (2)

After the construction of one-hot encodings for each category was completed, the original labels ranging from "1 to 11" across the 11 categories were mapped to their respective one-hot encoded vectors. The digit "1" was assigned the vector (1,0,0,0), the digit "5" was assigned the vector (1,1,0,0), and the subsequent categories were encoded in a similar manner, thus enabling the assembly of multi-label datasets.

### Data imbalance issues

The challenge of class imbalance within datasets involves a pronounced unevenness in the distribution of sample quantities across various categories, with some classes being markedly underrepresented compared to others [39]. Such a condition can exert a multifaceted impact on the training and efficacy of machine learning models:

1. Bias in Model Prediction: Models risk overfitting to the majority class, which can reduce their ability to accurately predict minority classes and decrease overall classification performance.
2. Misrepresentation of Evaluation Metrics: Relying solely on accuracy in imbalanced datasets can be misleading; a high accuracy might not reflect the model’s capability to identify minority classes, indicating poor classification effectiveness.
3. Alteration of Decision Boundaries: To maximize overall accuracy, models may shift decision boundaries favoring the majority class, leading to higher error rates for minority classes and affecting the model’s generalization.
4. Instability in Training and Prolonged Training Duration: Imbalanced data can cause models to converge to local rather than global optima, potentially requiring more training time and iterations to achieve a robust representation of all classes.

It becomes apparent that addressing the issue of data imbalance with suitable and potent methodologies is imperative prior to the development of a classification model. Prevalent strategies to counteract data imbalance encompass techniques such as oversampling, undersampling [40], cost-sensitive approaches, and ensemble learning to modulate the distribution of training data, thereby facilitating more robust learning and predictive capabilities for models across various classes.

The training dataset for this study exhibits extreme imbalance, with the ratio of the eleven lysine training samples, which consist of four PTMs—acetylation, crotonylation, methylation, and succinylation—being 9279:710:600:454: 561:251:360:88:153:454:73. Consequently, ClusterCentroids algorithm was selected to balance the dataset. An exhaustive exposition of this algorithm will be presented subsequently.

### Multi-LabelPosition-specificTripleAminoAcidPropensity,

#### MLPSTAAP

Based on Zuo’s research, the multi-label position-specific triad amino acid propensity (MLPSTAAP) algorithm was identified, which is designed for multi-class problems, demonstrates effective performance in the feature representation of protein sequences. This algorithm aligns well with the requirements of our study, and its specific steps are as follows:

Step 1: Calculate the frequency of each triad amino acid at each position within the lysine sequences of class t to obtain a matrix *F*_*t*_. The mathematical expression for this step is as follows:

$$F_{t}={\left[\begin{array}{cccc} F_{t}(TAA_{1}/1) & F_{t}(TAA_{1}/2) & \cdots & F_{t}(TAA_{1}/46)\\ F_{t}(TAA_{2}/1) & F_{t}(TAA_{2}/2) & \cdots & F_{t}(TAA_{2}/46)\\ \vdots & \vdots & \cdots & \vdots \\ F_{t}(TAA_{20^{3}}/1) & F_{t}(TAA_{20^{3}}/2) & \cdots & F_{t}(TAA_{20^{3}}/46) \end{array}\right]}_{20^{3}\times 46}$$ (3)

Where *t*∈{∅_1_,…,∅_11_},*F*_*t*_(*TAA*_*i*_/*j*) represents the frequency of the *TAA*_*i*_ triad amino acid at the j-th position in the lysine sequences of class t. It is straightforward to understand that there are 2^30^ riad amino acids *TAA*_*S*_ hence *TAA*_*i*_∈{*AAA*,*AAC*,*AAD*,…,*YYY*} where *i* = 1,2,…,20^3^, *j* = 1,2,3,…,46.

Step 2: Calculate the frequency at which each triad amino acid appears at each position in the lysine sequences of all classes except class t, to obtain the corresponding matrix *FF*_*t*_ for class t. The mathematical expression is as follows:

$$FF_{t}={\left[\begin{array}{cccc} FF_{t}(TAA_{1}/1) & FF_{t}(TAA_{1}/2) & \cdots & FF_{t}(TAA_{1}/46)\\ FF_{t}(TAA_{2}/1) & FF_{t}(TAA_{2}/2) & \cdots & FF_{t}(TAA_{2}/46)\\ \vdots & \vdots & \cdots & \vdots \\ FF_{t}(TAA_{20^{3}}/1) & FF_{t}(TAA_{20^{3}}/2) & \cdots & FF_{t}(TAA_{20^{3}}/46) \end{array}\right]}_{20^{3}\times 46}$$ (4)

Where $t=\emptyset_{1}\cup \ldots \cup \emptyset_{t-1}\cup \emptyset_{t+1}\cup \ldots \cup \emptyset_{11}.F_{t}({TAA}_{i}/j)$ represents the frequency of the triad amino acid *TAA*_*S*_ at the j-th position in the lysine sequences of the ten classes other than class t.

Step 3: Calculate the average of the obtained 11 *F*_*t*_ matrices and 11 *FF*_*t*_ matrices to obtain the matrices *F* and *FF*, respectively:

$$F=(F_{1}+F_{2}+{\ldots +F}_{11})/11$$ (5)

$$FF=({FF}_{1}+{FF}_{2}+{\ldots +FF}_{11})/11$$ (6)

And by subtracting the two matrices *F* and *FF*, the final feature matrix *F*_*MLPSTAAP*_. For a peptide segment containing 48 amino acids (omitting the central lysine (K)), the MLPSTAAP matrix can be represented as:

$$F_{MLPSTAAP}=F-FF={\left[\begin{array}{cccc} f_{1,1} & f_{1,2} & \cdots & f_{1,46}\\ f_{2,1} & f_{1,2} & \cdots & f_{2,46}\\ \vdots & \vdots & \cdots & \vdots \\ f_{20^{3},1} & f_{20^{3},2} & \cdots & f_{20^{3},46} \end{array}\right]}_{20^{3}\times 46}$$ (7)

$$Where\;f_{i,j}=F_{t}({TAA}_{i}/j)-{FF}_{t}({TAA}_{i}/j).$$

Step 4: The feature encoding is executed by identifying the values in the *F*_*MLPSTAAP*_ matrix corresponding to the tri-amino acid types at each position in the sequence. Taking a sample sequence "AGAT…" of length 48 as an example, a sliding window of size 3 is utilized to perform the encoding consecutively. Initially, the positions of tri-amino acid fragments within the sequence must be ascertained. For instance, "AGA" is located at the first position of the sequence, whereas "GAT" is at the second. Subsequently, the row position of each tri-amino acid in the matrix is calculated based on the amino acid alphabet ’A C D E F G H I K L M N P Q R S T V W Y’ $\left(row\;position=position1\times 20^{2}+position2\times 20^{1}+position3\times 20^{0}+1\right)$. In this alphabet, A, G, and T are positioned at the 0th, 5th, and 16th places, respectively. Thus, the row position for "AGA" is determined by the formula: 0×20^2^+5×20^1^+0×20^0^+1 = 101; similarly, the row position for "GAT" is calculated as: 5×20^2^+0×20^1^+16×20^0^+1 = 2017. Ultimately, the sequence is feature-encoded, with the first position of the feature vector being: *f*_101,1_, the second position being: *f*_2017,2_, and so on. A feature vector of length 46 is finally obtained.

#### ClusterCentroids undersampling algorithm framework

The ClusterCentroids algorithm, alternatively termed as the centroid-based clustering methodology, is classified under the umbrella of Prototype Generation (PG) algorithms. This particular PG algorithm initiates by ascertaining the equilibrium quantity of majority class samples predicated on the aggregate count of minority class samples. Subsequently, the K-means algorithm is harnessed to stochastically derive K centroids from the majority class samples. These computed centroids are then instantiated as novel representatives of the majority class, effectively supplanting the original samples and culminating in the realization of undersampling. A balanced representation is ensured by this strategic approach, conducive to an equitable model training environment.

The ClusterCentroids methodology adeptly harnesses clustering algorithms, such as K-means, to distill the majority class samples into a concise set of representative "centroids." These centroids, which encapsulate the core features of their respective clusters, are then repurposed as novel samples of the minority class. This elegant approach not only attenuates the overabundance of majority class samples but also retains the essential diversity of the dataset, adeptly addressing the challenge of class imbalance.

In the realm of clustering algorithms, K-means is renowned for its iterative refinement of centroids; however, its computational complexity escalates with dataset size, necessitating substantial memory resources. To mitigate this, our study employs MinibatchKmeans as the clustering algorithm within the undersampling process. As an enhanced variant of the traditional K-means, MinibatchKmeans enhances efficiency by operating on mini-batches of data to compute centroids. This approach not only expedites the clustering process and reduces memory consumption but also maintains a clustering performance marginally distinct from that of the conventional K-means algorithm. To validate this observation, the clustering time costs of both methods were compared on the dataset used in this study. A time cost of 15.66 seconds was recorded for K-means clustering, whereas only 10.61 seconds was required for MinibatchKmeans, indicating a significant advantage of MinibatchKmeans over the traditional K-means method. The pseudocode for MinibatchKmeans is presented in Box 2.

**Box 2. The pseudocode for MinibatchKmeans**

Input:

Dataset: D

Number of clusters: k

Mini-batch: b

Maximum number of iterations: T

Algorithm Procedure:

1. Randomly select k data points as initial cluster centers {*c*_1_,*c*_2_,…,*c*_*k*_}
2. Initialize counter t = 0
3. While t < T:
  - Randomly sample b instances from D to form a mini-batch dataset M.
  - For each instance *x*_*i*_ in M:
    - Compute the distance from *x*_*i*_ to each cluster centroid *c*_j_
    - Assign *x*_*i*_ to the nearest centroid
  - Update cluster centers:
    - For each cluster, calculate the mean of all mini-batch samples belonging to it
    - Update the centroids to these means.
  - t = t + 1

Output:

Cluster centers {*c*_1_,*c*_2_,…,*c*_*k*_}

Cluster labels for each sample

### CNN-based multi-label classifier

**Fig 4:**
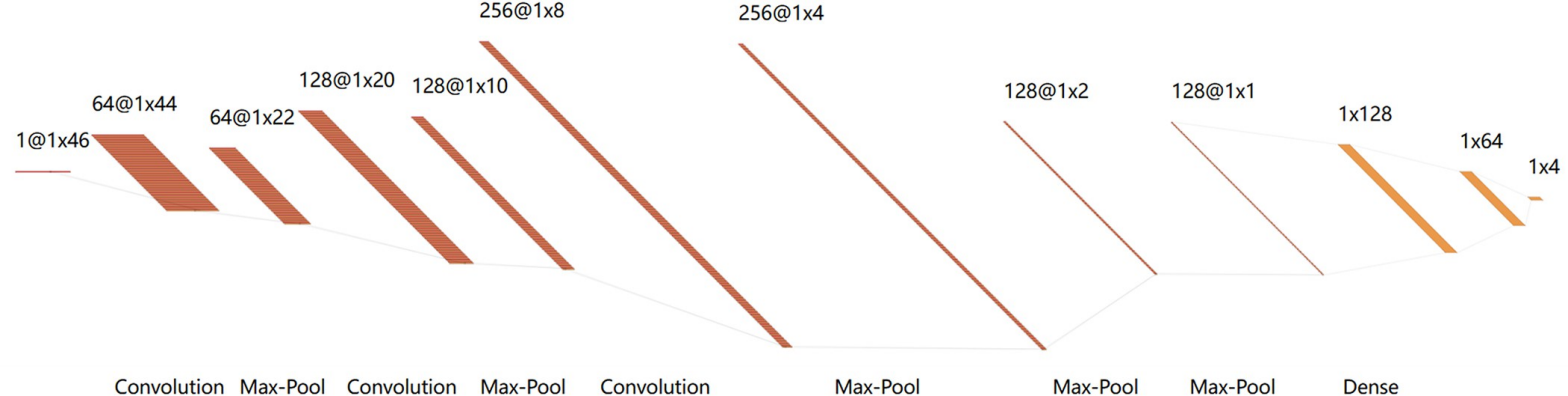
Neural network architecture diagram of this study.

Compared to traditional single-label classification, multi-label classification scenarios present a more complex and challenging task, as a sample may be associated with multiple category labels simultaneously [41]. To address the challenges associated with multi-label output in protein sequence site prediction, a classifier capable of effectively managing multi-label data is required. Protein sequence sites often exhibit strong correlations and class imbalance, which traditional dependency structures struggle to model adequately. Therefore, a deep learning framework based on Convolutional Neural Networks (CNNs) has been adopted, as illustrated in Fig 4, to better capture the rich information within sequences. The CNN architecture offers significant advantages for processing protein sequences: the initial convolutional layers, with their small receptive fields, are adept at extracting fine-grained local features such as key amino acid residues and their adjacent relationships, which are crucial for identifying functional regions within proteins. Concurrently, the hierarchical feature extraction mechanism of CNNs allows higher convolutional layers, with their larger receptive fields, to capture long-range dependencies and global patterns within the sequence. This multi-scale feature learning capability enables the CNN to comprehensively perceive the entire protein sequence and capture complex contextual and spatial structural information, thereby significantly enhancing the multi-label prediction performance for lysine modification sites.

Specifically, A sample data of size (1,46) was input into the classifier, comprising four CNN layers, four MAX-Pool layers, and one fully connected layer. The exponential linear unit (ReLU) is used as the activation function in the CNN layers:

ReLU(x)=max(0,x) (8)

The x represents the feature vector resulting from convolutional operations. In the output layer of the fully connected section, a sigmoid activation function is employed:

$$\sigma (x)=\frac{1}{1+e^{-x}}$$ (9)

The x denotes the (1,4) output matrix obtained after passing through the fully connected layer. This ensures that the outputted four-dimensional matrix lies within the range of 0 and 1, representing the model’s predicted probabilities for each of the four labels. The loss function utilized for training the classifier is the cross-entropy loss, which is suitable for classification problems [42]. The discrepancy between the target and predicted outputs is measured. The specific calculation formula is as follows:

$$BCE(y,\hat{y})=-\frac{1}{N}\sum_{i=1}^{N}\left[y_{i}\log\left({\hat{y}}_{i}\right)+(1-y_{i})\log\left(1-{\hat{y}}_{i}\right)\right]$$ (10)

Within this framework, N encapsulates the totality of samples under consideration, with *y*_*i*_ signifying the authentic label attributed to the i-th sample. Conversely, ${\hat{y}}_{i}$ corresponds to the probabilistic estimate rendered by the model for the i-th sample, essentially the output yielded by the model’s predictive machinery. In juxtaposition to other loss functions, such as mean squared error, a more substantial penalty on misclassifications is exerted by the binary cross-entropy loss, effectively nudging the model towards embracing accurate classification decisions with greater alacrity.

#### PreMLS construction

**Fig 5:**
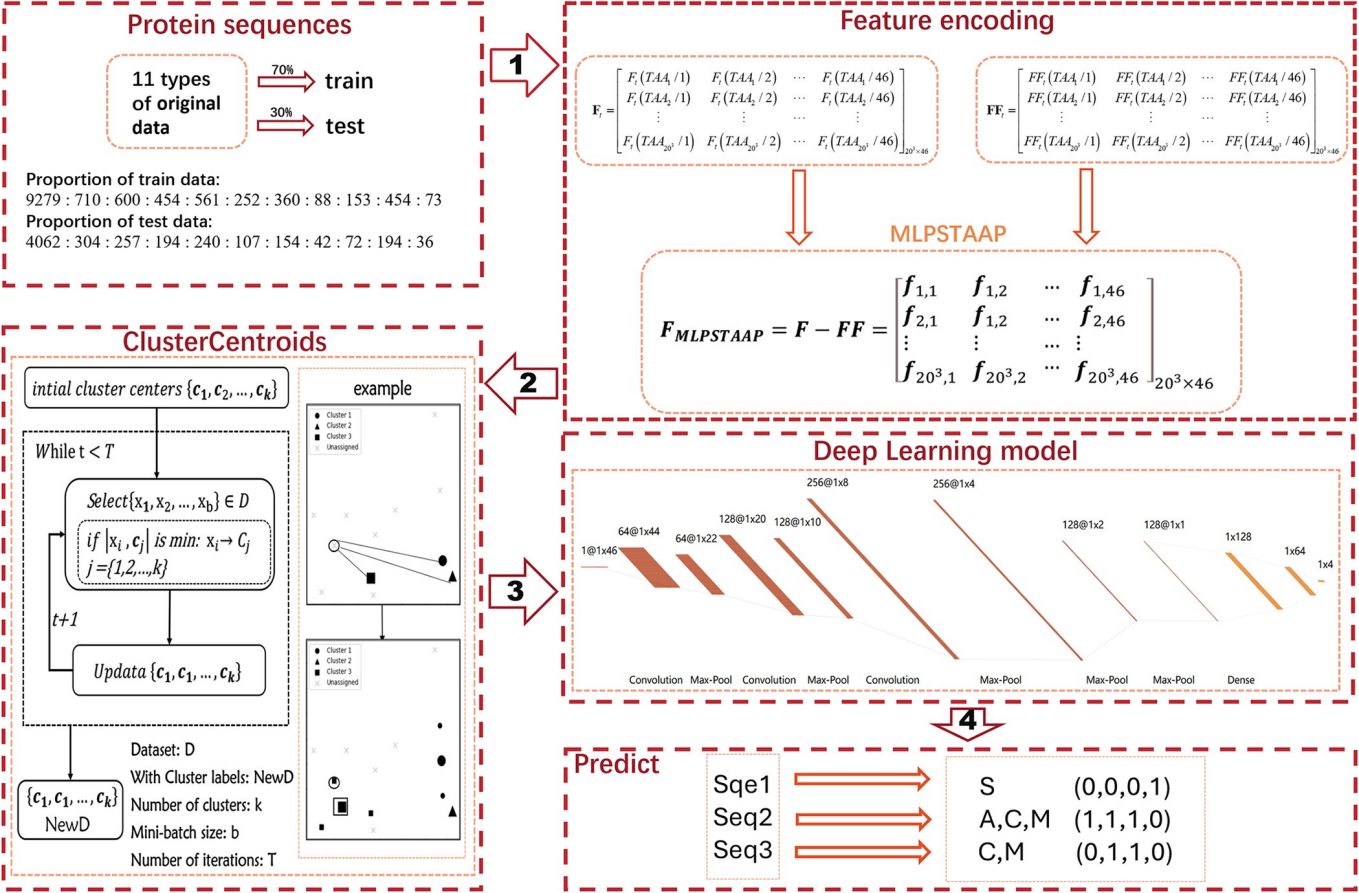
The flowchart of PreMLS constructed in this study.

In this study, PreMLS for multiple lysine site modifications has been developed, as schematically depicted in Fig 5. The construction process of PreMLS is as follows:

Step1: Through data collection, integration, and deduplication, we have amassed a dataset of protein sequences characterized by significant class imbalance, serving as our input data.

Step2: Utilizing a multi-label position-specific triad amino acid propensity feature extraction algorithm, we encoded the protein sequences to obtain a feature matrix.

Step3: Using the ClusterCentroids undersampling framework with MinibatchKmeans, the centroids of the majority classes were calculated, resulting in dataset undersampling. The subsampled data then served as the training dataset for the convolutional neural network.

Step4: The test data were propagated forward through the convolutional neural network to yield an output matrix. This matrix represents a total of 4,632 samples, with each sample corresponding to four label values. A threshold of 0.5 was set, where labels less than 0.5 were assigned a value of 0, and those equal to or greater than 0.5 were assigned a value of 1.


$$Pre(x)=\left\{ \begin{array}{l} 1,Output(x)\geq 0.5\\ 0,Output(x)<0.5 \end{array}\right.$$ (11)

Here, x denotes the model’s computed probabilities for the final predicted labels, with 0 signifying the exclusion and 1 indicating the inclusion of a label within a sample. The divergence between the output and target matrices is then ascertained through the loss function, which is employed to inform the backpropagation process and refine the parameters of the convolutional neural network.

#### Multi-label evaluation indicators

Following Chou’s research [43], Predictive Aiming (Aiming), Coverage, Accuracy, Absolute-True, and Absolute-False are employed in this study to assess the performance of various methods. These metrics are defined as follows:

$$Aiming=\frac{1}{n}\sum_{i=1}^{n}\left(\frac{\left|Y_{i}\cap {Y_{i}}^{*}\right|}{\left|{Y_{i}}^{*}\right|}\right)$$ (12)

$$Coverage=\frac{1}{n}\sum_{i=1}^{n}\left(\frac{\left|Y_{i}\cap {Y_{i}}^{*}\right|}{\left|Y_{i}\right|}\right)$$ (13)

$$Accuracy=\frac{1}{n}\sum_{i=1}^{n}\left(\frac{\left|Y_{i}\cap {Y_{i}}^{*}\right|}{\left|{{Y_{i}\cup Y}_{i}}^{*}\right|}\right)$$ (14)

$$Absolute-True=\frac{1}{n}\sum_{i=1}^{n}\Delta (Y_{i},{Y_{i}}^{*})$$ (15)

$$Absolute-False=\frac{1}{n}\sum_{i=1}^{n}\left(\frac{\left|Y_{i}\cup {Y_{i}}^{*}|-|Y_{i}\cap {Y_{i}}^{*}\right|}{M}\right)$$ (16)

With *n* representing the total sample number, *Y*_*i*_ as the actual labels, ${Y_{i}}^{*}$ as the predicted labels, and M as the total number of labels, these metrics provide a nuanced evaluation of the ability of PreMLS to accurately classify multi-label datasets.
